# SARS-CoV-2 RBD dimers elicit response comparable to VLPs in mice

**DOI:** 10.21203/rs.3.rs-2692315/v1

**Published:** 2023-04-28

**Authors:** J. Love, Sergio Rodriguez-Aponte, Lisa Tostanoski, Neil Dalvie, Ryan Johnston, Catherine Jacob-Dolan, Olivia Powers, Nicole Hachmann, Jessica Miller, Kevin Hall, Mazuba Siamatu, Camille Mazurek, Nehalee Surve, Dan Barouch

## Abstract

We report the direct comparison of monomeric, dimeric and trimeric RBD protein subunit vaccines to a virus-like particle (VLP) displaying RBD. After two and three doses, a RBD dimer and trimer elicited antibody levels in mice comparable to an RBD-VLP. Furthermore, an Omicron (BA.1) RBD hetero-dimer induced neutralizing activity similar to the RBD-VLP. A RBD hetero-dimer and RBD-VLP also shows comparable breadth to other SARS-CoV-2 variants-of-concern (VOCs).

## Main text

While global immunity to SARS-CoV-2 is increasingly widespread, continued manufacture and distribution of vaccines is necessary due to waning immunity after vaccination or infection[1]. Booster mRNA vaccines have been approved and distributed in high-income countries to protect against Omicron and other variants of concern (VOCs) that evade approved vaccines for COVID-19 matched to the original Wuhan-Hu-1 strain[2,3]. mRNA vaccines, however, are still not widely available in low- and middle-income countries (LMICs) due to high prices and limited supply[4]. Several protein subunit vaccines for COVID-19 based on the SARS-CoV-2 receptor binding domain (RBD) have been manufactured, approved, and widely distributed in LMICs[5]. Protein vaccines may be stored at higher temperatures for distribution in rural areas and manufactured in LMICs at low-cost using microbial hosts such as yeasts. Additional candidates for accessible booster vaccines could augment the expected need for new boosters to address the continued evolution of SARS-CoV-2.

Virus-like particles (VLPs) that multivalently display the RBD antigen can increase immunogenicity and protection compared to monomeric RBD in animal models[6,7] and humans[8]. Manufacturing of VLPs, however, is currently performed in mammalian cell hosts which increase production costs[9]. Indeed, the only currently approved RBD-VLP vaccine is produced with HEK-293 cells in Korea[8]. Several studies report that covalent or genetic multimerization of the RBD, like dimers and trimers, may improve immunogenicity relative to the monomeric RBD[10–13]. Some of these simple RBD multimers are currently under clinical evaluation[14,15]. Dimer- and trimer-based antigens could provide a modular platform for new variants. These could be designed and manufactured with updated serotypes to match existing Wuhan-Omicron boosters, for example. Moreover, they can leverage the low-cost manufacturing approaches for simpler antigens like monomeric RBDs. To our knowledge, there has not been a direct comparison between multimeric RBD vaccines and ones using VLP scaffolds. Here, we designed simple covalent multimers of the SARS-CoV-2 RBD protein that can be modularly manufactured in yeast[16,17]. We demonstrate that the immunogenicity of these multimers was improved related to the monomeric RBD, and was not significantly different from a previously reported RBD-VLP clinical candidate.

We sought to design simple covalent multimers of the SARS-CoV-2 RBD. We previously reported an RBD-VLP-based vaccine candidate conductive for manufacturing modularly in yeast[6], and we reasoned that a similar approach for covalent transpeptidation using SpyTag-SpyCatcher[18] could construct RBD dimers and trimers. We expressed genetic fusions of the RBD with SpyTag or SpyCatcher in yeast, and then covalently linked two or more complementary components by spontaneous overnight conjugation (Fig. 1a). This process yielded dimers and trimers of RBD or VLP's bearing RBD.  All RBD molecules contained two mutations (L452K+F490W) that, in our previous work, improved both the manufacturability and immunogenicity of the ancestral RBD molecule[19]; this engineered RBD also exhibited increased potency when displayed at high valency on a protein nanoparticle. We also constructed a heterotypic dimer of the RBD-L452K-F490W (RBD-J) and the Omicron (BA.1) RBD. Multimeric protein vaccines that include multiple RBD serotypes have exhibited increased breadth against SARS-CoV-2 VOCs[20,21]. We verified correct assembly of dimers, trimers, and VLPs by SDS-PAGE and biolayer interferometry (supplementary fig. S1). As expected, the dimers, trimers and VLPs showed increased avidity compared to the monomeric RBD. (supplementary fig. S1)

We evaluated the immunogenicity of each design in BALB/c mice by immunizing groups of five mice intramuscularly with 10μg of each RBD construct (Fig. 1a) formulated with 40μg of alum hydroxide. Separate groups of mice were immunized with either two doses at weeks 0 and 6, or three doses at weeks 0, 3, and 6. We collected and analyzed sera at weeks 0, 3, 6, 8, 10, and 24 (Fig. 1b).

After three weeks, we observed that a prime dose of the RBD-VLP elicited a potent response and high titers of antibodies against the WA1/2020 RBD. Mice immunized with a prime dose of RBD dimers (both homo- and hetero-dimer) and trimer showed lower, though not statistically different, levels of Spike-specific antibodies, compared to the RBD-VLP (Fig. 1c-d). The levels of Spike antibodies were significantly lower in mice immunized with a prime dose of either the RBD-monomer or SpyTag-RBD compared to the RBD-VLP at week 3.

A second dose of RBD led to seroconversion in all groups. At week 8 (after all doses), levels of Spike-specific antibodies were not significantly inferior between mice immunized with the RBD dimers or RBD trimer compared to mice immunized with the RBD-VLP, for mice that received either two or three doses (supplementary Table 1-2). The levels of Spike-specific antibodies in mice immunized with two doses of the RBD monomer or SpyTag-RBD monomer, however, remained significantly lower than the levels of antibodies in mice immunized with the RBD-VLP (supplementary Fig. 2, supplementary Table 1). When immunized with three doses, titers of Spike-specific antibodies in mice immunized with the RBD monomer were statistically comparable to the RBD-VLP, although we observed a higher variance in response in this group.

We observed a similar trend for Omicron BA.1 Spike-specific antibodies. Levels of Omicron BA.1 Spike antibodies in mice immunized with the RBD dimers or RBD trimer were not significantly inferior compared to mice immunized with the RBD-VLP, for mice that received either two or three doses (Fig. 1c-d). Levels of antibodies against the Omicron BA.1 Spike in mice immunized with two doses of either RBD monomer were significantly lower compared to the RBD-VLP at week 8 (supplementary Fig. 2a). Mice immunized with three doses of the RBD monomers exhibited statistically comparable levels of antibodies against Omicron BA.1 Spike compared to the RBD-VLP.

We then measured the titers of antibodies that neutralized the WA1/2020 or Omicron BA.1 SARS-CoV-2 pseudoviruses. When immunized with two doses of the RBD dimers and trimer, nAb titers elicited against the WA1/2020 or Omicron BA.1 pseudovirus were not significantly inferior compared to the RBD-VLP group at week 8 (Fig. 1e). In contrast, nAb titers against the WA1/2020 or Omicron BA.1 pseudoviruses elicited by two doses of either RBD monomer were significantly lower compared to the RBD-VLP, as expected (Fig. 1e). When immunized with three doses, nAb titers against the WA1/2020 pseudovirus elicited by the RBD monomers, dimers, and trimer were all statistically similar to the RBD-VLP at week 8 (Fig.1f). In mice immunized with three doses, nAb titers against the Omicron BA.1 were only significantly different from the RBD-VLP in mice immunized with the SpyTag-RBD (Fig. 1f). These results are consistent with reports suggesting that two vaccine doses elicit poor neutralization against Omicron BA.1, and that a third dose is required to have neutralizing activity in humans[22].

For all groups, Spike-binding antibodies were sustained through the end of the study at 24 weeks (Fig. 1c-d). Notably, neutralizing titers against the WA1/2020 and Omicron BA.1 pseudoviruses were also sustained for at least 6 months (Fig. 1g-h). The antibody levels and neutralizing antibody titers sustained in mice immunized with the RBD dimers and trimer were not significantly inferior to the RBD-VLP.

Overall, Spike-specific IgG levels had strong correlations with RBD-specific IgG levels for both WA1/2020 (Spearman R=0.9922, p<0.0001) and Omicron BA.1 (Spearman R=0.9904, p<0.0001) binding antibodies (Supplementary fig. 2a-b). Likewise, IgG levels had strong correlations with neutralizing titers (NT50) against the WA1/2020 (Spearman R=0.9281, p<0.0001) and Omicron BA.1 (Spearman R=0.7463, p<0.0001) pseudoviruses (Supplementary fig. 2c-d).

Lastly, we evaluated breadth against several SARS-CoV-2 VOCs. We observed that levels of Spike binding antibodies against VOCs from two doses of the RBD dimers and trimer were statistically comparable to the RBD-VLP (Fig. 2a, Supplementary Table 5). Two doses of the monomeric RBD groups exhibited substantially lower coverage to VOCs (Fig. 2a). After three doses, we observed that the RBD dimers and trimer elicited similar coverage to VOCs compared to the RBD-VLP (Fig. 2b, Supplementary Table 6). In this case, the Omicron (BA.1) Spike-specific IgG response elicited by the hetero-dimer was highest among the three dose groups, although not significantly higher. Finally, we evaluated the durability of this response to more novel VOCs, such as Omicron sub-variants. We observed that RBD dimers, trimer and VLP sustained comparable levels of Spike protein specific antibodies at week 24 (Fig. 2c-d). Notably, the RBD hetero-dimer sustained the highest median IgG levels to Omicron sub-variants (Fig. 2d), although not significantly different from the RBD homo-dimer, trimer and VLP.

In this study, we have demonstrated that RBD dimers can elicit comparable responses to RBD-VLPs in mice after two or three doses with a conventional adjuvant (alum). These results support the utility of RBD dimers to elicit strong responses and breadth against SARS-CoV-2 variants. In particular, a hetero-dimeric presentation of RBDs including the Omicron BA.1 RBD, showed a slightly enhanced response against VOCs. Given the evolving viral landscape[23], future studies should focus on evaluating these RBD dimers as booster-only alternatives in animal models with preexisting immunity. These RBD dimers can be modified or mixed to present RBD combinations that may lead to increased coverage to VOCs. Furthermore, additional studies with novel adjuvants[24] could inform approaches to further potentiate the neutralizing response and durability of dimer-based antigens.

Additionally, questions remain about the durability of neutralizing responses to novel variants[2] afforded by currently approved vaccines to SARS-CoV-2. Alternative formulations for boosters could rely on candidates amenable to low-cost, scalable and transferable manufacturing that is accessible globally. RBD dimers could also serve as a multivalent display platform for novel RBD variants either in heterotypic or cocktail forms[21]. Such candidates may also have utility for a low-cost seasonal[25] solution to decaying immunity and emerging variants.

### Methods

## Strains

Recombinant genes for RBD variant expression were codon optimized for *Komagataella phaffii* expression, and synthesized cloned into a custom vector on a BioXP (Telesis Bio). Linear DNA was purified, and constructs were transformed into a modified wild-type *K. phaffii* (NRRL Y-11430) as described previously[19].

## Cultivations

Strains producing material for further analytical characterization and mice immunization were cultivated in 400mL in 2L baffled flasks as described previously[19]. Cells were inoculated at 0.1 $OD_{600}$, grown for 24 hours in complex media containing 4% glycerol, pelleted, resuspended, and grown for 24 hours in complex media containing 40 g/L sorbitol and 1.5% methanol.

## Protein Purification

Purification of harvested cell culture supernatant containing the recombinant protein was performed using the downstream processing (DSP) module of InSCyT as described previously[26]. The supernatant pH was adjusted inline to pH 5.0 with 100mM citric acid. The supernatant was loaded into a 5mL prepacked CMM HyperCel column (Sartorius AG, Gottingen, Germany). The column was re-equilibrated with 20mM sodium citrate, pH 5.0, washed with 20mM sodium phosphate pH 6.5, and elution was initiated with 20mM sodium phosphate, 300mM NaCl, pH 8.5. Eluate from column 1 above 20mAu was loaded into a 1mL prepacked HyperCel STAR AX column (Sartorius AG, Gottingen, Germany). Flow-through material above 20mAu was collected.

## Sodium dodecyl sulfate polyacrylamide gel electrophoresis (SDS-PAGE)

SDS-PAGE for protein characterization and conjugation confirmation was performed as previously described[6]. Samples were diluted with Novex™ 2X Tris-glycine sample buffer (Invitrogen, LC2676), and

NuPAGE™ 10X sample reducing agent (Invitrogen, NP0009), and heated at 95˚C for 15 min. Approximately 0.1 µg of each sample was loaded on 12% Tris-glycine gel (Invitrogen, WXP01220BOX) run for 90 min at 140 V in 1X Tris-glycine SDS running buffer (Invitrogen, LC2675). Novex™ PageRuler pre-stained protein ladder (Invitrogen, 26616) was used as marker to estimate molecular weight of the proteins. The gel was stained using InstantBlue® Coomassie Protein Stain (Abcam, ab119211) for 60 minutes and destained with water for at least another 60 minutes

## Biolayer interferometry (BLI)

Biolayer interferometry was performed using the Octet Red96 with Protein A (ProA) biosensors (Sartorius ForteBio, Fremont, CA) as previously described[26]. ProA biosensors were hydrated for 20 min in kinetics buffer prior to each run. Kinetics buffer comprising 1X PBS pH 7.2, 0.5% BSA, and 0.05% Tween 20 was used for all dilutions, baseline, and disassociation steps. ACE2-Fc was loaded at a concentration of 10 µg/mL. Samples were loaded in a 96-well black microplate (Greiner Bio-One, Monroe, NC) at starting concentrations of 15 µg/mL for ACE2-Fc binding. Association and dissociation were measured at 1000 rpm for 300 and 600 seconds, respectively. Binding affinity was calculated using the Octet Data Analysis software v10.0 (Pall ForteBio), using reference subtraction, baseline alignment, inter-step correction, Savitzky-Golay filtering, and a global 1:1 binding model.

## Preparation of vaccine materials

Purified RBD-SpyTag was conjugated onto Hepatitis B surface antigen (HBsAg) SpyCatcher VLPs overnight at a 1.2:1 RBD to HBsAg molar ratio. For RBD dimers, RBD-SpyTag and RBD-SpyCatcher were conjugated overnight at a 1:1.2 molar ratio. For RBD trimers, SpyTag-RBD-SpyTag and RBD-SpyCatcher were conjugated overnight at a 1:2.5 molar ratio. Conjugated RBD-VLP was buffer exchanged and concentrated with 100K molecular weight cutoff Pierce™ protein concentrators (Thermo Fisher, 88524). Conjugated RBD dimers and trimers were buffer exchanged and concentrated using 50K molecular weight cutoff Pierce™ protein concentrators (Thermo Fisher, 88539). RBD monomer and RBD-SpyTag were buffer exchanged and concentrated using 10K molecular weight cutoff Pierce™ protein concentrators (Thermo Fisher, 88517).

Materials were formulated with a 10mM Histidine, 20mM Sodium phosphate, 5mM Tris, 37.5mM NaCl, pH 7.4 buffer. All RBD groups were diluted to a final total protein concentration of 100µg/mL. Alhydrogel (Invivogen, vac-alu-250) was added to a final concentration of 400µg/mL.

## Animals and study design

Female C57BL/6 mice (The Jackson Laboratory) 4–6 weeks of age were randomly assigned to experimental groups. Mice were immunized with 10µg of the indicated RBD formulation, adjuvanted with 40µg of alum hydroxide, via intramuscular injection (n = 5/group). Separate groups of mice were administered either a two-dose regimen, with injections at weeks 0 and 6, or a three-dose regimen, with injections at weeks 0, 3, and 6. At the indicated timepoints, peripheral blood was collected via the submandibular route and processed to isolate serum for immunologic or virologic assays. All animal studies were conducted in compliance with relevant local, state, and federal guidelines. All animal studies were reviewed and approved by the Beth Israel Deaconess Medical Center Institutional Animal Care and Use Committee.

*Electrochemiluminescence assay (ECLA)* ECLA plates (MesoScale Discovery SARS-CoV-2 IgG, Panels 22, 23) were designed and produced for multiplex binding assays with up to 10 antigen spots in each well, including either Spike or RBD proteins from multiple SARS-CoV-2 variants. Plates were blocked with 150µL of Blocker A solution (1% bovine serum albumin (BSA) in distilled water) for at least 30m at room temperature with shaking at 700 rpm on a digital microplate shaker. The plates were then washed 3 times with 150µL of wash buffer (0.5% Tween in 1x PBS), blotted dry, and 50µL of serum samples, pre-diluted 1:5,000 in Diluent 100 (MesoScale Discovery), were added in duplicate and the plates were set to incubate with shaking at 700rpm at room temperature for 2h. Secondary antibody was prepared using Jackson Immuno Rabbit Anti-Mouse IgG detection antibody (Cat No: 315-005-045) conjugated to the MSD GOLD SULFO-TAG by NHS-Ester chemistry per the manufacturer's guidelines (Cat No: R91AO-1). The plates were washed 3 times and 50µL of tagged secondary antibody diluted 1:500 in Diluent 100 was added to each well and incubated shaking at 700 rpm at room temperature for 1 h. Plates were then washed 3 times and 150µL of MSD GOLD Read Buffer B (MesoScale Discovery) was added to each well and the plates were read immediately after on a MESO QuickPlex SQ 120 machine. Antibody levels for each sample were reported as Relative Light Units (RLU) which were calculated as the average Sample RLU minus Blank RLU for each sample. 2x106 RLU was defined as the upper limit of detection. For samples with signal exceeding this value, samples were pre-diluted further, assays were repeated; the resultant RLU value was multiplied by the additional dilution factor to correct for this dilution before analysis. 1000 RLU was defined as the lower limit of detection for each assay.

## Pseudovirus neutralization

The SARS-CoV-2 pseudotyped viruses were generated using human embryonic kidney (HEK) 293T cells (American Type Culture Collection CRL_3216) which were co-transfected with a spike protein expressing pcDNA3.1-SARS-CoV2 SΔCT (WA1/2020 strain (Wuhan/WIV04/2019, Global Initiative on Sharing Avian Influenza Data (GISAID) accession ID: EPI_ISL_402124, B.1.1.529 [Omicron BA.1, GISAID ID: EPI_ISL_7358094.2], and BA.4/BA.5 [GSAID ID: EPI_ISL_12268495.2]), a packaging plasmid psPAX2 (AIDS Resource and Reagent program), and the luciferase reporter pLenti-cytomegalovirus Puro-Luc (Addgene). 48 hours post co-transfection, supernatants containing pseudoviruses were collected. To measure neutralizing activity of the serum, HEK293T-human angiotensin-converting enzyme 2 (hACE2) cells were seeded at a density of 2.0 × 104 in 96-well tissue culture plates overnight. Threefold serial dilutions of heat-inactivated serum samples were prepared and mixed with 60 µl of pseudovirus and incubated at 37°C for 1 hour. This mixture was then added to the seeded HEK293T-hACE2 cells, and 48 hours later, Steady-Glo Luciferase Assay (Promega) was used to lyse cells according to the manufacturer's instructions. SARS-CoV-2 neutralization titers were determined as the sample dilution at which a 50% reduction (NT50) in relative light units (RLUs) was observed relative to the average of the virus control wells.

## Declarations

### Acknowledgements

This work was funded by the Bill and Melinda Gates Foundation (Investment IDs INV-027406 (D.H.B.), INV-041469 (D.H.B.), and INV-002740 (J.C.L)). This study was also supported in part by the Koch Institute Support (core) Grant P30-CA14051 from the National Cancer Institute. The following reagent was deposited by the Centers for Disease Control and Prevention and obtained through BEI Resources, NIAID, NIH: SARS-Related Coronavirus 2, Isolate USA-WA1/2020, NR-53780. L.H.T. is an NIH T32 Postdoctoral Fellow supported by the Multidisciplinary AIDS Training Program (Grant # T32 AI007387). The content is solely the responsibility of the authors and does not necessarily represent the official views of the NCI, the NIH, or the Bill and Melinda Gates Foundation.

### Author contributions

S.R.A, L.H.T., N.C.D, D.H.B., and J.C.L. conceived and planned experiments. S.R.A analyzed unformulated drug substance. L.H.T. coordinated and conducted animal experiments with assistance from C.J.D. and O.C.P. L.H.T., C.J.D., O.C.P., and K.H. performed immunologic assays. O.C.P., N.P.H., J.M., K.H., M.S., C.R.M., and N.S. performed virologic assays. S.R.A, L.H.T., D.H.B., and J.C.L. wrote the manuscript. J.C.L and D.H.B. designed the experimental strategy and reviewed analyses of data. All authors reviewed the manuscript.

### Competing interests

S.R.A., N.C.D., and J.C.L. have filed a patent related to the RBD-L452K-F490W (RBD-J) sequence. J.C.L. has interests in Sunflower Therapeutics PBC, Honeycomb Biotechnologies, OneCyte Biotechnologies, QuantumCyte, and Repligen. J.C.L's interests are reviewed and managed under MIT's policies for potential conflicts of interest.

## Figures

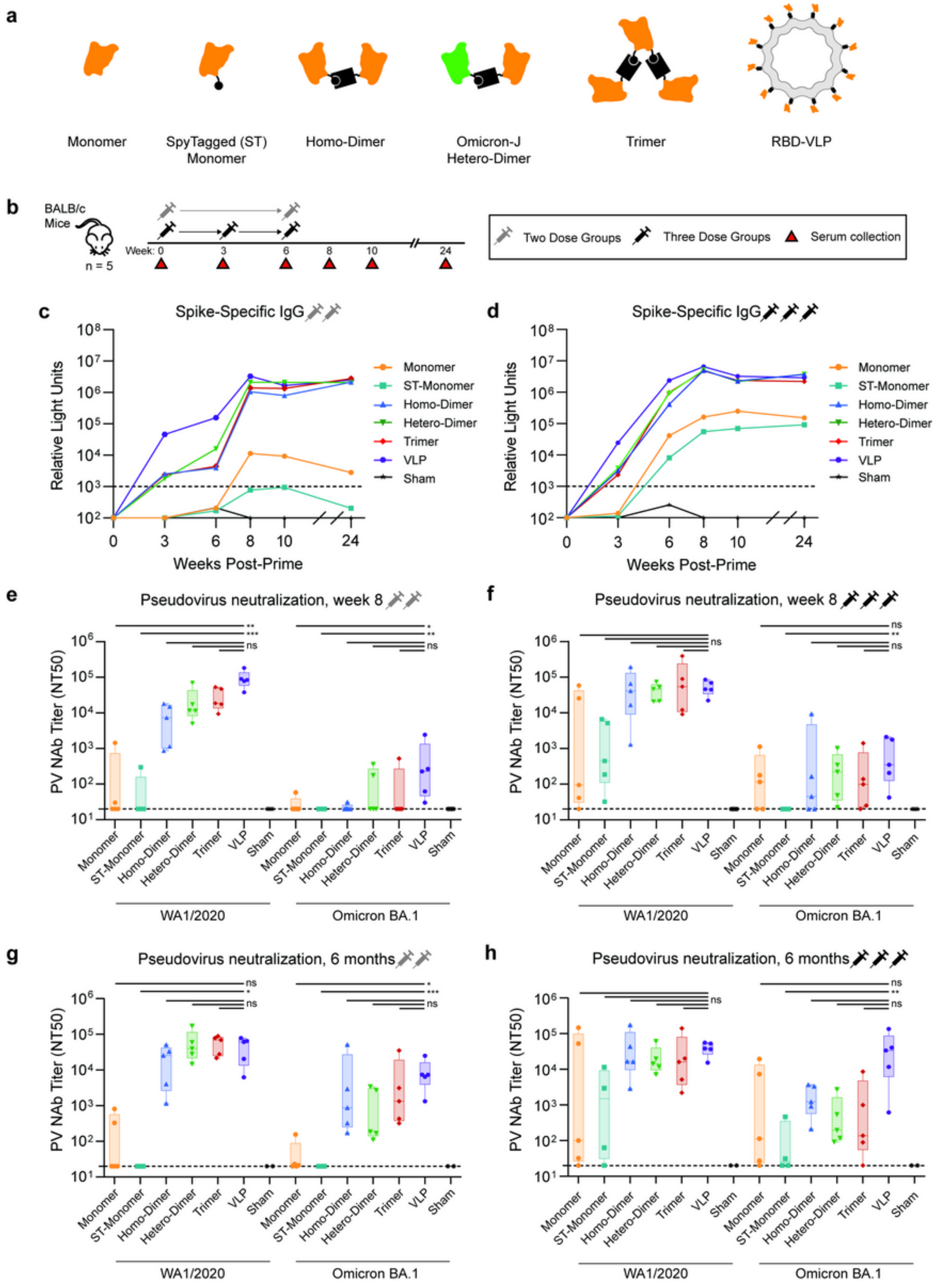

### Figure 1

**Humoral response to RBD oligomers and VLP**. (a) Design of RBD groups presented as a monomer, dimer, trimer and VLP. Orange RBDs indicate the use of RBD-J, and green RBDs indicate the use of Omicron BA.1. (b) Design of mice study using BALB/c mice. (c-d) WA1/2020 Spike-specific antibody levels in mice sera in (c) two dose and (d) three dose groups. Plotted values indicate the median IgG titer. (e-f) Titer of neutralizing antibodies against WA1/2020 and Omicron (BA.1) SARS-CoV-2 pseudoviruses in mice sera of (e) two dose and (f) three dose groups at week 8. (g-h) Titer of neutralizing antibodies against WA1/2020 and Omicron (BA.1) SARS-CoV-2 pseudoviruses in mice sera of (g) two dose and (h) three dose groups at week 8. Bars indicate the median NT50 response. Significance was determined by a Kruskal-Wallis test using Dunn's multiple comparison method (*p<0.05, **p<0.01, ***p<0.001, ****p<0.001).

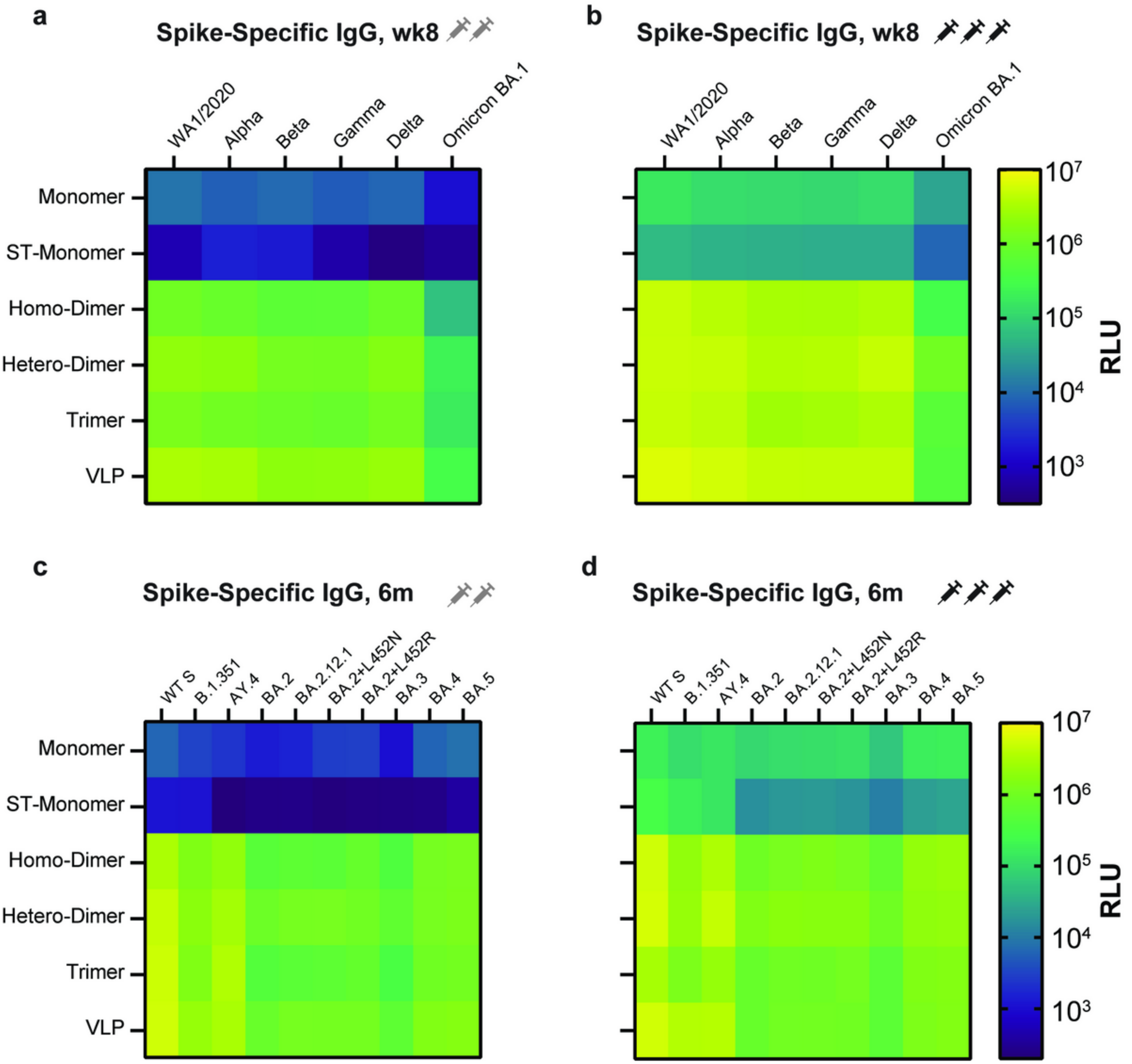

### Figure 2

**Antibody titers against SARS-CoV-2 VOCs**. (a-b) Antibody levels of VOCs Spike-specific antibodies in mice sera in (a) two dose and (b) three dose groups at week 8. (c-d) Antibody levels of VOCs Spike proteinspecific antibodies in mice sera of (c) two dose and (d) three dose groups at 6 months. Plotted values indicate the median IgG response.

## Supplementary Files

This is a list of supplementary files associated with this preprint. Click to download.

- FigS1.tif
- FigS2.tif
- SupTable1.tif
- SupTable2.tif
- SupTable3.tif
- SupTable4.tif
- SupTable5.tif
- SupTable6.tif
